# Meta-Analytic Structural Equation Modeling of the Influences of Family-Centered Care on Parent and Child Psychological Health

**DOI:** 10.1155/2009/576840

**Published:** 2009-11-30

**Authors:** Carl J. Dunst, Carol M. Trivette

**Affiliations:** ^1^Orelena Hawks Puckett Institute, 8 Elk Mountain Road, Asheville, NC 28804, USA; ^2^Orelena Hawks Puckett Institute, 128 South Sterling Street, Morganton, NC 28655, USA

## Abstract

*Background*. Family-centered care is now practiced throughout the world by physicians, nurses, and allied health care professionals. The call for adoption of family-centered care is based on the contention that the physical and psychological health of a child is influenced by parents' psychological health where family-centered care enhances parent well-being which in turn influences child well-being. We empirically assessed whether these relationships are supported by available evidence. *Method*. Meta-analytic structural equation modeling was used to test the direct and indirect influences of family-centered care and self-efficacy beliefs on parent and child psychological health. Data from more than 2900 parents and other caregivers in 15 studies were used for the analyses. *Results*. Family-centered care had indirect effects on parent and child psychological health mediated by self-efficacy beliefs. *Conclusion*. The relationships posited in the literature about family-centered care were supported by the study results.

## 1. Introduction

Family-centered care is defined as an innovative approach to planning, delivering, and evaluating health care to children and adolescents grounded in mutually beneficial partnerships and collaborations between health care professionals and families [1]. It is practiced by treating families with dignity and respect, information sharing so families are fully knowledgeable about their children's condition and care, family participation in both decision-making and the health care of their children, and a working alliance between health care professionals and family members [2–4]. Family-centered care is how health care professionals interact, treat, and involve patients' family members in their care and treatment. 

Family-centered care has increasingly been adopted by hospitals, physicians, nurses, and allied health professionals as a way of improving health care to children [5]. This approach to help giving is now practiced in many countries, including, but not limited to, Australia, Canada, England, Finland, Iceland, Ireland, Russia, Sweden, South Africa, The Netherlands, New Zealand, and the United States [6–13]. The practice has been endorsed or recommended by both professional and international organizations [14–16]. The call for adoption of family-centered care is based, in part, on the contention that the physical and psychological health of a child is likely influenced by parents' psychological health where family-centered care enhances parent well-being which in turn influences child well-being [17, 18]. Fifty years ago, the Platt report included the recommendation that parents should be involved in the care of their hospitalized children, and that the emotional needs of both the parents and children must be addressed so that the benefits of health care could be maximized [19].

There is a considerable amount of evidence that family-centered care is related to parents' enhanced psychological health [20–22]. There is also evidence that family-centered care is indirectly related to children's psychological health mediated by self-efficacy beliefs [20]. There are no studies to the best of our knowledge that have investigated the direct and indirect effects of family-centered care on both parents' and children's psychological health where the hypothesized relationships linking parents' health to children's health have been empirically examined. However, qualitative studies of family-centered care include descriptions of practices and parent and child behavior which indicate that family-centered care may affect both parent and child psychological health [14, 23, 24].

This paper includes the findings from a research synthesis using meta-analytic structural equation modeling for testing the direct and indirect effects of family-centered care on parent and child psychological health to ascertain if the relationships posited in the literature are supported by empirical evidence. Meta-analysis (MA) is a procedure for combining results from different studies and assessing whether the combined strength of the relationships between variables are sufficiently large to claim a causal or functional relationship between an independent variable and dependent variables of interest [25]. Structural equation modeling (SEM) is a procedure for building a causal model, hypothesizing the nature of the relationships between the variables in the model, and testing whether the model fits the patterns of relationships among measures [26]. Meta-analytic structural equation modeling (MASEM) uses data from different studies and combines the data to produce a pooled correlation or covariance matrix, where the pooled matrix is used to test an SEM model [27, 28].

**Figure 1:**
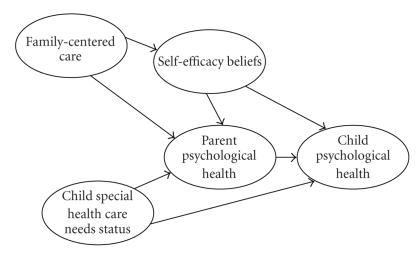
Structural equation model for depicting the effects of family-centered care, self-efficacy beliefs, and child special health care needs on parent and child psychological health.

Figure 1 shows the model that guided our MASEM. The model includes family-centered care [2], parental self-efficacy beliefs [29], parent psychological health [30], child psychological health [31], and child special health care needs [32]. Family-centered care was hypothesized to have direct effects on both parent self-efficacy beliefs and parent psychological health, and indirect effects on parent psychological health mediated by self-efficacy beliefs. These causal paths are based on findings from meta-analyses of the relationships between family-centered practices, self-efficacy beliefs, and parent behavior and functioning [20, 21]. Self-efficacy beliefs were hypothesized to have direct effects on both parent and child psychological health, and indirect effects on child health mediated by parent psychological health. These causal paths are based on research on the relationships between self-efficacy beliefs and parent and child behavior and functioning [20, 33]. Parent psychological well-being was expected to directly affect child health based on research demonstrating a relationship between parent and child affective behavior [34, 35]. More complex special health care needs were expected to be negatively related to both parent [36] and child [37] psychological health based on research demonstrating the consequences of the birth and rearing of a child with special needs [38, 39].

Family-centered care was measured in terms of relational and participatory help giving practices [40]. Relational family-centered practices include behavior typically associated with effective clinical skills (active listening, compassion, respect, etc.) and professional positive attributions about family strengths and capabilities [23]. Participatory family-centered practices include information sharing so families can make informed choices, family involvement in acting on those choices, and professional flexibility and responsiveness to family requests [41]. Self-efficacy beliefs were measured in terms of parents' control appraisals of the ways professionals treated their families and their perceived control over important life events [42]. Parents control appraisals of professional behavior include the belief that one can obtain advice and support when needed, and control appraisals over life events include the belief that one can execute a course of action to produce desired consequences. Findings from different studies show that family-centered practices influence control appraisals of how professionals treat families which in turn contributes to a general sense of perceived control over other life events [33, 43]. Parent and child psychological health were both measured in terms of positive and negative well-being [44, 45]. Indicators of parent and child positive psychological health include behavioral expressions of joy, elation, calmness, and so forth; whereas indicators of negative psychological health include behavioral expressions of sadness, anxiety, sleep difficulties, frustration, and so forth. Special health care needs status was measured in terms of the presence of a disability or identified medical condition that increased the need for health care beyond that which would be typical for most children [46].

## 2. Materials and Methods

### 2.1. MASEM Studies

Fifteen studies conducted by ourselves and our colleagues were used for the MASEM. The data for the analyses came from a mix of studies published in refereed journals [43, 47–51], two monographs [20, 33], and a book chapter [52], as well as three unpublished studies [53–55].

The criteria for selecting studies for the MASEM were the inclusion of measures of family-centered practices, self-efficacy beliefs, either or both parent psychological health and child psychological health, and child special health care need status in the same study. An extensive review of the published and unpublished literature located no studies other than our own that included self-efficacy belief measures or included the correlations among measures needed to perform a MASEM [20, 21]. The largest majority of studies of family-centered care include measures of parent satisfaction [21] rather than self-efficacy beliefs and we have determined that satisfaction is not an adequate proxy for these belief appraisals [20].

### 2.2. Study Participants

The 15 studies included 2948 parents and other primary caregivers. Most were mothers (94%) and were married or living with a partner (82%). The participants were, on average, 33 years of age (range = 17 to 67) and completed 14 years of formal education (range = 5 to 25). The majority of the participants were Caucasian (93%) while the others were Black (4%), Latino (2%), or another race or ethnicity (1%). The socioeconomic status of the participants' families varied from low to high.

The participants' children were, on average, 39 months of age (range = 3 to 172). Forty six percent of the children were males and 54% were females. Half of the children had an identified disability or diagnosis associated with the need for special health care (46%) while the other half had developmental delays without any identified condition or diagnosis (54%). Information about children's diagnoses and developmental delays was obtained from medical records and multidisciplinary team evaluations, or the results of developmental tests when information from the other two sources was not available. The children's diagnoses were made by pediatricians, family physicians, and professionals from specialty care centers, developmental evaluation programs, and early intervention programs.

### 2.3. Measures

The study participants completed a battery of scales about themselves, their children, and the professionals with whom they were working. This included measures of family-centered care, self-efficacy beliefs, and parent and child psychological health. Information available on the children was used to code child health care status.

The family-centered care measures included the *Helpgiving Practices Scale* [56], *Family-Centered Practices Scale* [57], *Enabling Practices Scale* [58], and a modified version of the *Family-Centered Practices Scale* [59]. Each of the scales included items measuring both relational and participatory family-centered practices. The scales were completed by each child's parent or caregiver who were asked to indicate the extent to which the help-giver working most closely with their family interacted and treated them and their child in ways consistent with family-centered scale indicators. Separate analyses of the relational and participatory practices items on the scales in each study all produced single factor solutions [60].

The self-efficacy belief measures included the *Personal Assessment of Control Scale* [61], *Practitioner Personal Control Scale* [62], *Early Intervention Efficacy Appraisal Scale* [63], and *Degree of Personal Influences Scale* [64]. Each of the scales measure either or both perceived control over the help and assistance provided by a professional working with the family and perceived control over other life events.

Parent psychological health was measured by the *Center for Epidemiological Studies Depression Scale* [65], *Psychological Well-Being Scale* [66], *Personal Health and Well-Being Scale* [67], and one investigator developed measure. All the scales included indicators of positive and negative health. Separate analyses of the two sets of items in each study produced single factor solutions [60].

The child psychological health measures included selected items on both the *Carolina Record of Individual Behavior* [68] and the *Child Learning Opportunities Scale* [69]. Both instruments include indicators of positive and negative child affect. The psychometric analyses of the two sets of items in each study produced single factor solutions.

Special health care needs status was first ascertained by dividing children in the individual studies into two groups: (1) developmental delays without any diagnosis or medical reasons for the delays and (2) identified disabilities and associated medical concerns (e.g., low birth weight, prematurity). Each group was further divided into two subgroups. The children with developmental delays were assigned to either a domain-specific developmental delay (e.g., language) or a global delay in multiple areas group. The children with identified disabilities and medical concerns were assigned to either an identified condition without any secondary concerns or a multiple disability/medical concern group. Orthogonal contrast coding [70] was used to place the children on a continuum from a domain specific delay to multiple disability/medical concerns for data analysis purposes. A higher score indicated more complex special health care needs.

### 2.4. Methods of Analysis

A two-stage, four step meta-analytic structural equation modeling procedure [71] was used to produce a pooled correlation matrix from the data in the 15 studies and to use the pooled matrix to perform the structural equation model analysis. The first step involved a test of the homogeneity of the correlation matrices from the individual studies. The patterns of correlations among the variables in the different studies need to be relatively similar in order to produce a pooled matrix. The second step is to obtain a weighted pooled correlation matrix. This involves adjustments to the strength of the relationships between variables by giving more weight to studies with larger sample sizes. The third step is to conduct a confirmatory factor analysis to ascertain if measured variables used to construct latent variables (e.g., family-centered care = relational + participatory practices) are justified. The fourth step is to fit the hypothesized model (Figure 1) to the pooled correlation matrix to test the fit of the structural equation model to the data.

At the different steps, goodness of fit statistics are used to determine if required assumptions are met. Two fit indices were used in the analyses: Comparative fit index (CFI) and root mean square error of approximation (RMSEA). CFI ranges from zero to 1, where a value of 0.90 or higher is considered an index of acceptable fit to the data. (The closer CFI is to 1.00, the better the fit.) RMSEA ranges from zero to 1, where a value of 0.05 or less is considered an acceptable fit. (The closer RMSEA is to zero, the better the fit.) All analyses were performed using LISREL [72].

Two SEMs were tested. The first model treated family-centered care, self-efficacy beliefs, and parent and child psychological health as latent variables where each was assumed to have two measured variables (relational and participatory practices; professional and life events control; parent positive and negative health; child positive and negative health). The second model treated professional and life events control as separate measured variables based on previous research showing that family-centered care influences professional control appraisals which in turn influences life events control [43].

Both the direct and indirect effects of family-centered care and self-efficacy beliefs on parent and child psychological health were examined as part of the SEMs. Direct effects are estimated statistically by the path coefficients (parameter estimates) between two measured or latent variables. Indirect effects are estimated by the product of two direct effects (e.g., the indirect effects of family-centered care on parent psychological health mediated by self-efficacy beliefs are estimated by the product of the path coefficients between family-centered care and self-efficacy beliefs, and self-efficacy beliefs and parent psychological health). The sizes of the direct and indirect effects were assessed by standardized path coefficients which can range between −1.00 and 1.00.

The SEM was performed by the weighted least squares method with the weighted correlation matrix (Table 1) as the input [72]. The signs of the negative parent and child psychological health measures were reversed for the analyses to avoid artifactual suppression [73].

## 3. Results

### 3.1. Homogeneity of the Correlation Matrices

This is a test of whether the correlation matrices in the 15 different studies can be assumed to be derived from the same population. CFI was 0.91 and RMSEA was 0.09. The results indicate that the different correlation matrices were reasonably similar to produce a pooled correlation matrix.

### 3.2. Pooled Correlation Matrix

Table 1 shows the weighted pooled correlation matrix. The correlations between variables across studies were combined by weighted averages giving more weight to studies with larger sample sizes and by taking into consideration other statistical artifacts [27, 74].

The largest majority of correlations are statistically significant because of the combined large sample size (*N* = 2948) in the 15 studies. Relational and participatory family-centered practices were highly related to each other, and both were related to all the other measures except parent negative psychological health. The two self-efficacy measures were related to each other, and both were related to all the other measures except child negative psychological health. Parent positive and negative psychological health were related to one another, but differentially related to child psychological health and child special health care status albeit in the opposite way expected. (The more complex the children's special health care needs, the better were the parents' judgments of the children's psychological health.) The two child psychological health measures were only minimally related to each other, but both were related to child special health care status.

### 3.3. Confirmatory Factor Analysis

The confirmatory factor analysis (CFA) assessed the extent to which the measured variables for family-centered care, self-efficacy beliefs, parent psychological health, and child psychological health each produced a single factor solution. The CFA included child special health care status as a separate measured variable. CFI was 1.00 and RMSEA was 0.04, indicating a good fit of the CFA model to the data. Notwithstanding the confirmatory factor analysis results, the factor loadings on the self-efficacy, parent psychological health, and child psychological health latent variables were dissimilar, indicating that the measured variables differentially contributed to the relationships among measures. For example, the factor loadings for self-efficacy beliefs were 1.00 for control over professional behavior and 0.31 for control over life events. This is reflected by the fact that the two family-centered practices measures are more strongly related to professional control compared to life events control (Table 1). This pattern of results was the basis, in part, for proposing and conducting the respecified SEM introduced previously and described in detail below.

### 3.4. Structural Equation Model Findings

**Figure 2:**
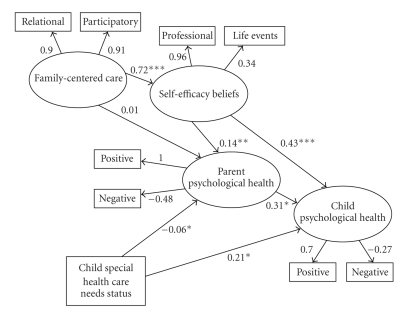
Structural equation model results for the effects of family-centered care, self-efficacy beliefs, and child special health care needs on parent and child psychological health (MODEL I). (Note: the significance levels of the path coefficients are influenced by the standard errors for those metrics and are the reason why larger coefficients sometimes have smaller *P*-values.) *P < .01, **P < .001, ***P < .0001.

The first model tested was the one in Figure 1 with family-centered care, self-efficacy beliefs, parent psychological health, and child psychological health as latent variables with each having two measured variables (see Table 1) and child special health care status as a separate measured variable. The results are shown in Figure 2. CFI was 1.00 and RMSEA was 0.04. These indices show a good fit of the model to the data.

As predicted, family-centered care was directly related to self-efficacy beliefs (*B* = 0.72, *P* < .0001), and self-efficacy beliefs were in turn directly related to both parent and child psychological health (*B*s = 0.14 and 0.43, *P*s < .001 and .0001, resp.). The more professionals were judged as family centered, the stronger the participants' self-efficacy beliefs, and the stronger the parents' self-efficacy beliefs; the more positive and less negative were parent and child psychological health. Family-centered care was also indirectly related to both parent psychological health (*B* = 0.10, *P* < .05) and child psychological health (*B* = 0.31, *P* < .001) mediated by self-efficacy beliefs. The more family centered were professional practices, the more positive and less negative were parent and child psychological health.

As expected, self-efficacy beliefs were directly related to both parent and child psychological health (*B*s = .14 and .43, *P*s <.001 and .0001), but not indirectly related to child psychological health mediated by parent psychological health as predicted. The stronger the participants' self-efficacy beliefs, the more positive and less negative were parent and child psychological health. Parent psychological health was directly related to child psychological health as predicted (*B* = 0.31, *P* < .01). Child special health care status had a small negative effect on parent psychological health (*B* = .06, *P* < .05) but was positively related to child psychological health (*B* = 0.21, *P* < .01). Contrary to expectation, the more complex the children's special health care needs, the more positive and less negative was child psychological health.

Despite the fact that the MASEM results for the first model were consistent with the hypothesized relationships among variables, close examination of Figure 2 indicates that the relationships between self-efficacy beliefs and parent and child psychological health may have been suppressed [73] by the fact that family-centered care was differentially related to the two self-efficacy belief measures (Table 1). The respecified model permitted an assessment of whether this in fact was the case.

### 3.5. Respecified Structural Equation Model Findings

**Figure 3:**
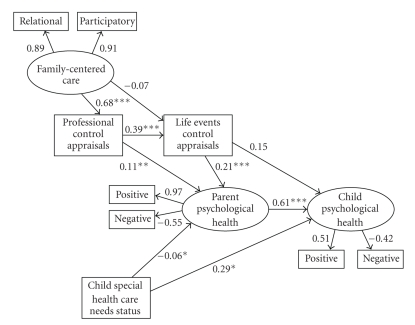
Structural equation modeling results for the respecified model (MODEL II). *P < .01, **P < .001, ***P < .0001.

Figure 3 shows the respecified model. CFI was 1.00 and RMSEA was 0.04. The pattern of relationships among the measures was as hypothesized, and as suspected, treating self-efficacy beliefs as a latent variable in fact suppressed the effects between belief appraisals and parent and child psychological health.

Family-centered care had a direct effect on control over professional family-centered practices (*B* = 0.68, *P* < .0001) and an indirect effect on control over life events mediated by professional control (*B* = 0.27, *P* < .001). The more family centered were professional practices, the stronger the participants' self-efficacy beliefs.

Control over professional family-centered practices had a direct effect on parent psychological health (*B* = 0.11, *P* < .01) and an indirect effect on parent psychological health mediated by life events control (*B* = 0.08, *P* < .05). Control over life events had direct effects on both parent (*B* = 0.21, *P* < .01) and child (*B* = 0.15, *P* < .01) psychological health and an indirect effect on child health mediated by parent health (*B* = 0.13, *P* < .001). In all cases, the stronger the participants' self-efficacy beliefs, the more positive and less negative were parent and child psychological health.

Child special health care needs had a small negative direct effect on parent psychological health and a direct positive effect on child psychological health. The more complex the children's special health care needs, the more attenuated was the parents' psychological health but the more positive was the children's psychological health.

## 4. Discussion

The manner in which family-centered care and self-efficacy beliefs were related to parent and child psychological health was as hypothesized. Family-centered care had direct effects on self-efficacy beliefs and indirect effects on parent psychological health mediated by belief appraisals. Self-efficacy beliefs had direct effects on parent and child psychological health and indirect effects on child health medicated by parent health. The patterns of results provide support for the contention that family-centered care influences parent psychological health which in turn influences child psychological health [24]. The findings add to our understanding of effects of family-centered care by demonstrating the role self-efficacy beliefs play in affecting parent and child psychological health.

The reason self-efficacy beliefs rather than a construct like patient or family satisfaction is a preferred mediator is made clear when one considers the target of study participant appraisals. Satisfaction is a measure of someone else's behavior [75]; whereas self-efficacy is a measure of one's own beliefs about executing a course of action to produce a desired or expected result [29]. Many years of research has shown that self-efficacy beliefs affect people's behavior in many domains of functioning [29, 42, 76]. As part of a meta-analysis of family-centered practice research, we compared the indirect effects of family-centered care on parent, family, and child behavior mediated by both self-efficacy beliefs and satisfaction, and in every analysis found belief appraisals a much stronger mediating variable [20].

The one unexpected finding was the relationship between child special health care status and child psychological health. Contrary to expectation, children with more complex health care needs were judged as having better psychological health by their parents. The reason why this was the case is not immediately apparent. The results point to a need to further investigate the relationship between children's backgrounds and conditions and their psychological health to find explanations for this counterintuitive result. Post hoc examination of the data in the studies constituting the focus of analysis yielded no hints for why special health care status was associated with better child psychological health.

This study has both strengths and limitations. The strengths include the use of both meta-analysis and structural equation modeling for testing the direct and indirect effects of family-centered care on parent and child psychological health. The limitations include the fact that all the study measures were self-report scales which may have contributed to artifactual covariation among measures. This limitation is partly offset by the fact that in other studies where parent and child positive and negative well-being were obtained by observational measures, the same relationships found in the study reported in this paper were found in those studies [77].

Several other limitations should also be mentioned. One is the fact that all the studies were conducted by ourselves and our colleagues with children and families primarily in two states in the USA. The extent to which the findings can be generalized to families in other states and other countries awaits replication. Another limitation could be a publication bias since studies that are likely to report positive results are more likely to be published; whereas studies that yield no appreciable relationship among variables are less likely to be published. This limitation is partly offset by the fact that the studies included in the MASEM included a mix of published and unpublished studies and were included based on the measures used in the studies and not the relationships among the measures.

Because most of the measures constituting the focus of investigation in the studies in the MASEM were collected at the same time, the direction of influence of the variable may be different or even opposite than those that were hypothesized. This, however, is not likely to be the case since in those studies where family-centered care was measured at one time and psychological health was measured at a later time, the relationships among the measures were much the same regardless of when the measures were taken [43, 51].

## 5. Conclusion

The relationships posited in the literature between family-centered care and parent and child psychological health were supported by the study results with the caveat that the influences are mostly indirect rather than direct. The findings advance our understanding of these relationships by showing how family-centered care is indirectly related to parent and child psychological health mediated by self-efficacy beliefs.

## Figures and Tables

**Table 1:** Weighted pooled correlation matrix for the relationships between the study variables.

Measures	Family-centered care		Self-efficacy beliefs		Parent health		Child health		SH
	RP	PP	PC	LC	PH	NH	CP	CN	
*Family-centered care*									
Relational practices (RPs)	—	.82***	.61***	.13***	.11***	−.05**	.34***	−.14***	−.04*
Participatory practices (PPs)		—	.62***	.14***	.10***	−.01	.32***	−.07**	−.06**

*Self-efficacy beliefs*									
Professional control (PC)			—	.30***	.16***	−.05**	.28***	−.02	−.04*
Life events control (LC)				—	.23***	−.20***	−.09***	−.02	.09***

*Parent psychological health*									
Positive health (PH)					—	−.53***	.19***	−.24***	−.06**
Negative health (NH)						—	−.02	.21***	.01

*Child psychological health*									
Positive health (PH)							—	−.21***	.09***
Negative health (NH)								—	−.14***

*Child special health (SH) care needs*									—

*P < .05, **P < .01, ***P < .0001.
